# Supplementary figures and images for: Exogenous 2-(3,4-Dichlorophenoxy) triethylamine ameliorates the soil drought effect on nitrogen metabolism in maize during the pre-female inflorescence emergence stage
Source: BMC Plant Biol. 2019 Mar 19;19:107. doi: 10.1186/s12870-019-1710-5 (PMC6425708; doi:10.1186/s12870-019-1710-5)

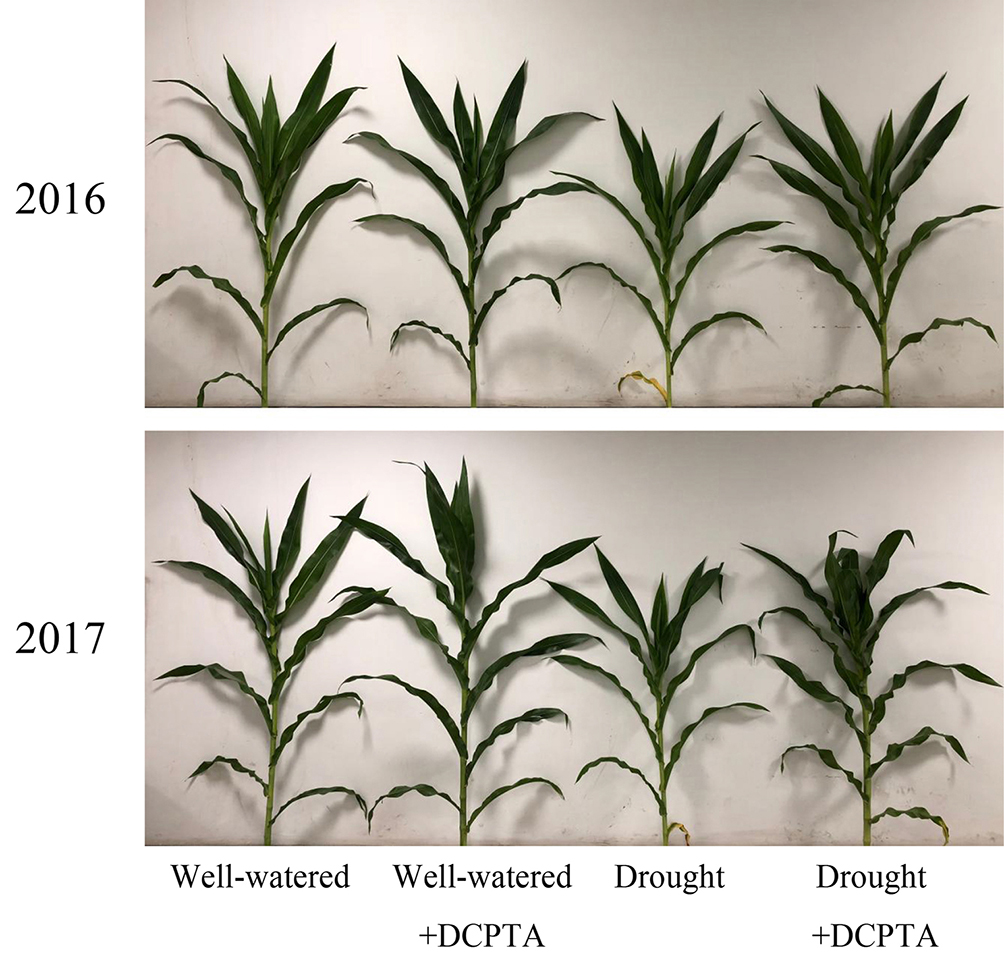

Supplement: Supplementary file 1 — Leaf phenotypic features of the maize seedlings after 30 days of treatment with drought and/or DCPTA in 2016 and 2017. (JPG 531 kb) [file 12870_2019_1710_MOESM1_ESM.jpg]

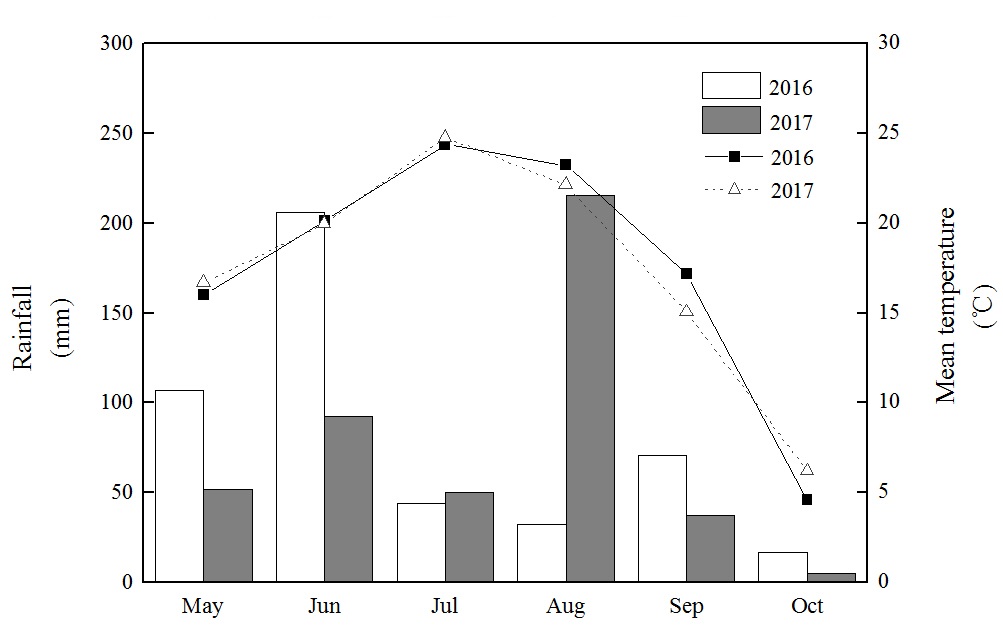

Supplement: Supplementary file 2 — The rainfall (bar) and mean temperature (line) data during the study period (2016 and 2017, May–October). (JPG 68 kb) [file 12870_2019_1710_MOESM2_ESM.jpg]

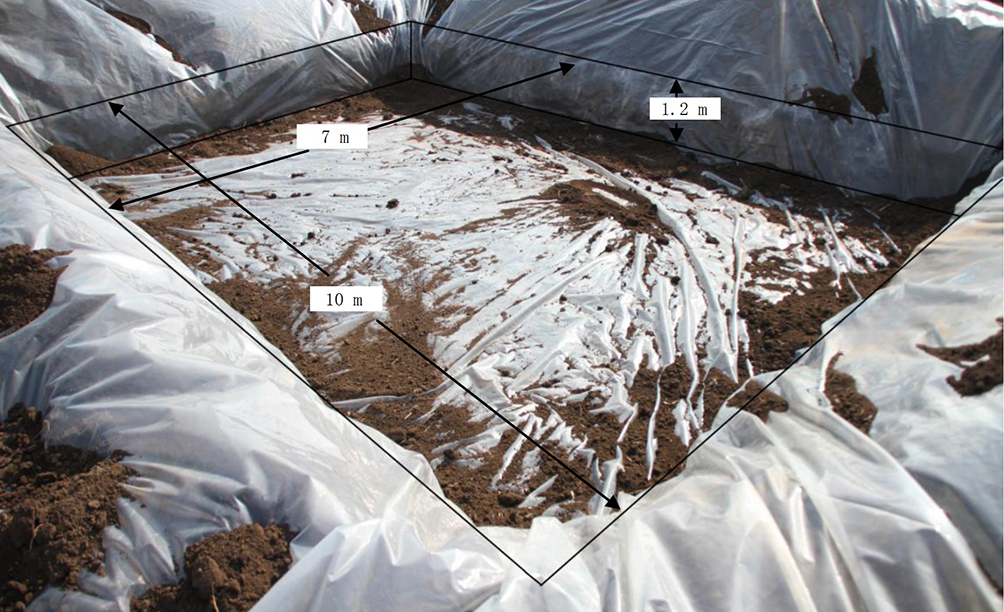

Supplement: Supplementary file 3 — The pits (inner length, 10 m; width, 7 m; and height, 1.2 m) used for this study and the plastic sheets used to cover inner sides of the pits. (JPG 513 kb) [file 12870_2019_1710_MOESM3_ESM.jpg]

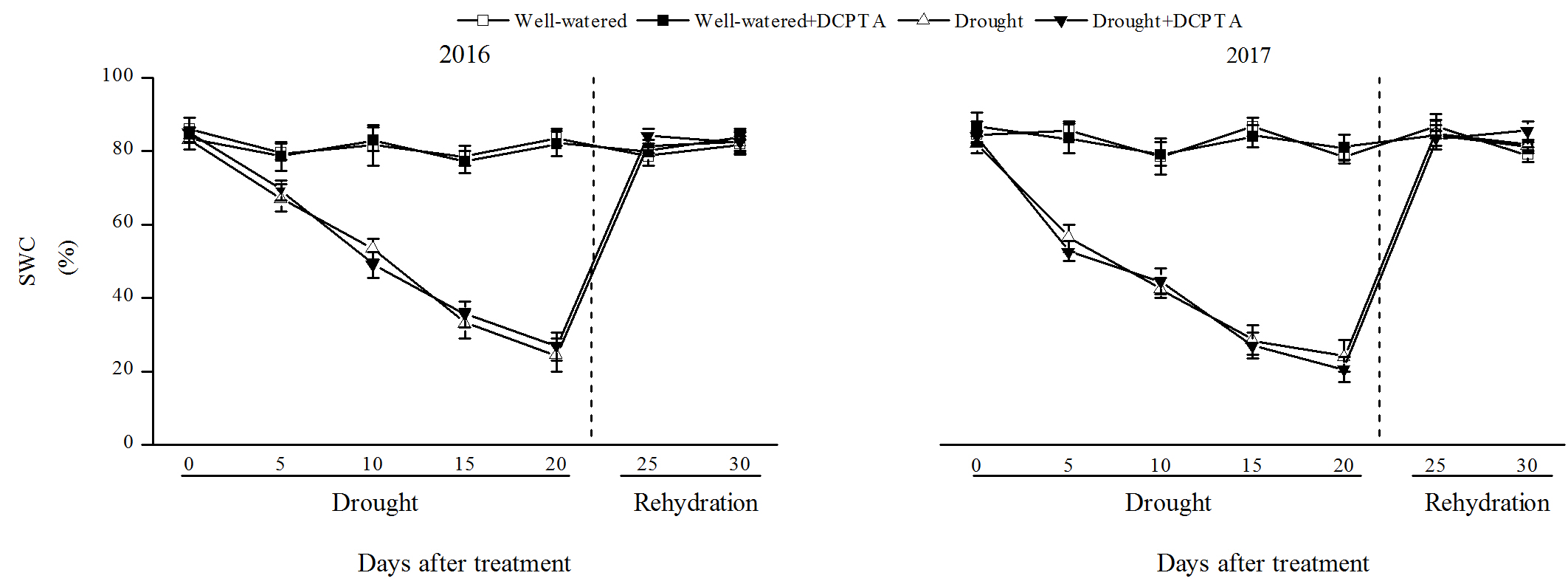

Supplement: Supplementary file 4 — Changes in the soil water content (SWC) in 2016 and 2017. The data represent the means of independent measurements with five replicates, and the standard deviations are indicated by the vertical error bars. Values with the same letters on the bars are not significantly different at P < 0.05 (LSD test). (JPG 325 kb) [file 12870_2019_1710_MOESM4_ESM.jpg]

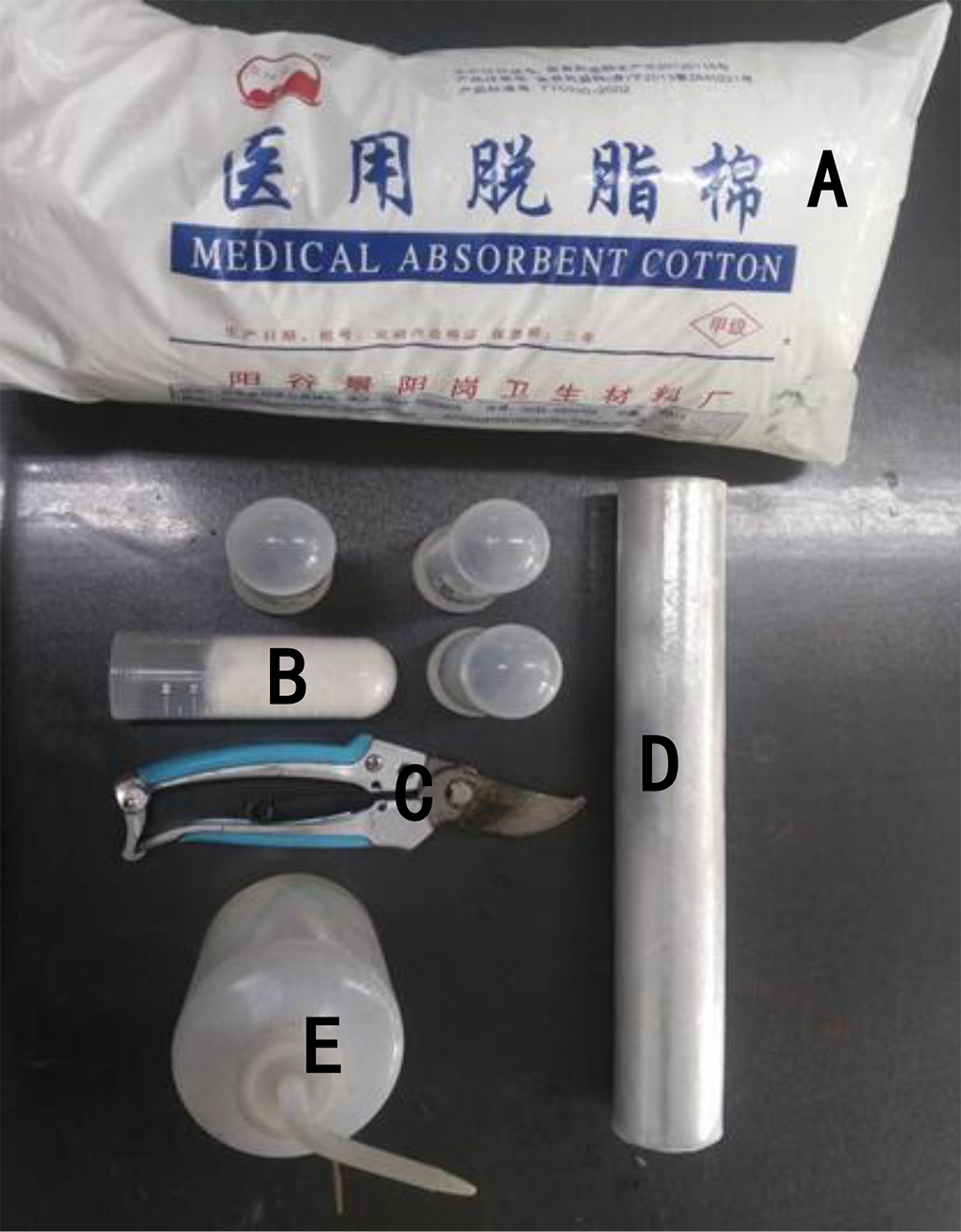

Supplement: Supplementary file 5 — The tools used for the collection of bleeding sap. Cotton (A), centrifuge tube (B), scissors (C), plastic film (D), and deionized water (E). (JPG 471 kb) [file 12870_2019_1710_MOESM5_ESM.jpg]
